# Increased Proteoglycanases in Pulmonary Valves after Birth Correlate with Extracellular Matrix Maturation and Valve Sculpting

**DOI:** 10.3390/jcdd10010027

**Published:** 2023-01-11

**Authors:** Loren E. Dupuis, Sarah E. Evins, Morgan C. Abell, Morgan E. Blakley, Samuel L. Horkey, Jeremy L. Barth, Christine B. Kern

**Affiliations:** Department of Regenerative Medicine and Cell Biology, Medical University of South Carolina, Charleston, SC 29425, USA

**Keywords:** ADAMTS, MMP, proteoglycanase, cardiac valve, pulmonary valve, mechanotransduction, versican, decorin, ADAMTS5

## Abstract

Increased mechanical forces on developing cardiac valves drive formation of the highly organized extracellular matrix (ECM) providing tissue integrity and promoting cell behavior and signaling. However, the ability to investigate the response of cardiac valve cells to increased mechanical forces is challenging and remains poorly understood. The developmental window from birth (P0) to postnatal day 7 (P7) when biomechanical forces on the pulmonary valve (PV) are altered due to the initiation of blood flow to the lungs was evaluated in this study. Grossly enlarged PV, in mice deficient in the proteoglycan protease ADAMTS5, exhibited a transient phenotypic rescue from postnatal day 0 (P0) to P7; the *Adamts5^−/−^* aortic valves (AV) did not exhibit a phenotypic correction. We hypothesized that blood flow, initiated to the lungs at birth, alters mechanical load on the PV and promotes ECM maturation. In the *Adamts5^−/−^* PV, there was an increase in localization of the proteoglycan proteases ADAMTS1, MMP2, and MMP9 that correlated with reduced Versican (VCAN). At birth, Decorin (DCN), a Collagen I binding, small leucine-rich proteoglycan, exhibited complementary stratified localization to VCAN in the wild type at P0 but colocalized with VCAN in *Adamts5^−/−^* PV; concomitant with the phenotypic rescue at P7, the PVs in *Adamts5^−/−^* mice exhibited stratification of VCAN and DCN similar to wild type. This study indicates that increased mechanical forces on the PV at birth may activate ECM proteases to organize specialized ECM layers during cardiac valve maturation.

## 1. Introduction

Adult cardiac valves comprise a specialized, stratified extracellular matrix (ECM) that gives structural support to ensure unidirectional blood flow. In addition to imparting the mechanical properties of tissues, the ECM instructs cell behavior and signaling [1,2]. The reciprocal interactions of cells and ECM are driven by biomechanical forces. However, mechanisms that integrate cell-matrix responses with mechanical load are not well understood. 

The provisional ECM within endocardial cushions, the precursors of the cardiac valves, is rich in hyaluronic acid and the aggregating proteoglycan Versican (VCAN). This early ECM provides an environment conducive to cell migration and proliferation. As the embryo grows and mechanical forces are increased, the provisional ECM is remodeled by synthesis of fibrillar collagens, assembly of elastin, and proteolytic cleavage and clearance of VCAN [3]. The mature ECM components become stratified within the developing cusp (also referred to as leaflet) while the cusp morphology changes from block shaped to sculpted. Previous studies have revealed that the valvular endocardium senses changes in mechanical forces and propagates the signals to underlying valvular interstitial cells (VICs, specialized fibroblasts) through multiple mechanotransductive pathways [4,5]. Although cardiac valve ECM mirrors the biomechanical forces it endures, the ECM factors activated in response to mechanotransductive pathways remain elusive and challenging to investigate [1,6].

Transitioning of the ECM requires ECM proteases and their cleaved substrates which are components of the degradome, a relatively understudied subset of the ECM proteome. Activation of ECM proteases can elicit a transformative impact on the ECM architecture through proteolytic cleavage of their ECM substrates for bioassembly as well as activation, degradation, and clearance [7]. Cleavage of the aggregating proteoglycans VCAN and Aggrecan (ACAN) also generates bioactive fragments referred to as Versikine [8] and Aggrekine [9,10], respectively. Use of mouse models deficient in ECM proteoglycan proteases (i.e., proteoglycanases) together with neo-epitope specific antibodies that identify the corresponding cleaved ECM substrates [11,12,13] has uncovered critical in vivo ECM proteolytic events emphasizing the importance of the degradome [14,15,16,17,18,19,20,21,22]. Specifically with respect to cardiac valve development, mice deficient in A Disintegrin and Metalloproteinase with Thrombospondin motifs-5 (ADAMTS5) or ADAMTS9 proteoglycanases exhibit enlarged malformed cardiac valves due to excess of their proteoglycan substrates VCAN and ACAN [14,23]; the VCAN and ACAN proteolytic profiles, as identified by neo-epitope specific antibodies, are also disrupted [8,9,10]. In vivo reduction of VCAN rescues the enlarged ADAMTS5-deficient (*Adamts5^−/−^*) valve phenotype, indicating that cleavage and clearance of VCAN are essential for cardiac valve maturation [14]. There is also evidence that mutations in ADAMTS5 and ADAMTS19 contribute to cardiac valve anomalies and disease in the patient population [24,25,26]; however, the ECM substrates of ADAMTS19 have not been identified. Since excess proteoglycans are associated with human cardiac valve disease and aortopathies [27,28,29,30], determining how proteoglycan content is controlled by ECM proteoglycanases in response to mechanical load may be important for therapeutic and regenerative approaches to treat cardiac valve diseases.

This study focuses on our observation that the grossly enlarged PVs in mice deficient in the proteoglycan protease ADAMTS5, exhibited a transient phenotypic rescue from P0 to P7; the *Adamts5^−/−^* aortic valves (AV) did not exhibit significant morphological changes during this timeframe. Shortly after birth, the closure of the ductus arteriosus and foramen ovale routes blood to the lungs through the PV, thereby increasing its mechanical load. Coincident with birth, ECM proteoglycanases ADAMTS1, MMP2, and MMP9 exhibited increased expression profiles in the in the PVs of *Adamts5^−/−^* mice, indicating a potential to compensate for the loss of ADAMTS5. These data indicated that ECM proteoglycanases may be amplified in response to increased mechanical load at birth to promote specialized ECM formation during postnatal cardiac valve maturation.

## 2. Materials and Methods

### 2.1. Valve Cusp and Hinge Quantification

To quantify the cusps and hinge regions of the PV as well as the AV, histological sections over a depth of 30 μM were quantified for each heart; i.e., the widest portion of the anterior, right, and left cusps of the PV, and the right coronary, left coronary, and non coronary cusps of the AV were measured. The narrowest part of the hinge regions was also measured and quantified. Measurements were averaged then used for graphs and statistical analysis. Of note, hearts that exhibited a bicuspid phenotype or had other cardiac anomalies such as a ventricular septal defect were not utilized in this study. Amira™ 3D 2021.1 (Visage Imaging, Andover, MA, USA) was used to generate three dimensional (3D) reconstructions from 5 μm-thick paraffin sections. For each P0 or P7 reconstruction, approximately 100–140 sections per valve were used. 

### 2.2. Immunohistochemistry

Standard histological procedures were used [31]. Decorin (DCN) antibody (AF1060) was purchased from R & D Systems Inc. Antibodies to α-smooth muscle actin (SMA) (Sigma, A 5228) were used to identify smooth muscle cells. Fluor-conjugated secondary antibodies were purchased from Jackson ImmunoResearch (West Grove, PA, USA). Antibodies were used in murine tissues fixed in Amsterdam (Amst: 5% methanol, 35% acetone, 5% acetic acid) [14] or 4% paraformaldehyde (para). All para-fixed tissue was treated with either citric acid antigen unmasking (H-3300, Vector laboratories, Burlingame, CA, USA) for ECM antibodies to murine VCAN (gift from Dr. S. Hoffman) and DCN or proteinase K treatment for ADAMTS1 (SC5468), MMP2 (AB19167) and MMP9 (AB38898) localization. Post-fixation involved 80% ethanol for 5 min, followed by 50% ethanol for 5 min, and then two rinses in phosphate-buffered saline. Imaging was performed on the Leica TCS SP5 or SP8 AOBS Confocal Microscope System (Leica Microsystems Inc., Exton, PA, USA). Images in panels are an example of a minimum of three different experimental replicas of *Adamts5^−/−^* and littermate controls (*Adamts5^+/+^*; wild type—WT). Digital images of *Adamts5*^−/−^ and WT heart sections were acquired at identical confocal settings using the Leica TCS SP5 or SP8 AOBS Confocal Microscope System. 

### 2.3. Statistics

For quantification of the valve cusp and hinge width, data were analyzed using GraphPad Prism 9.1 (GraphPad Software Inc.). A one-way analysis of variance (Anova) or the nonparametric Kruskal–Wallis test was used to determine the differences between the groups wild type (+/+) and *Adamts5^−/−^* (−/−), at timepoints, P0, P7 and 1 mo. When Anova assumptions of normality and equal variances could not be met, a Kruskal–Wallis test was used to compare groups. Adjusted *p*-values are indicated for each group comparison, with ⍺ = 0.05. Anova and Kruskal–Wallis tests revealed significant differences between groups. GraphPad was utilized to generate graphs. In graphs, each symbol represents data from a single mouse (n = 1) for evaluation of cardiac valve cusp and hinge regions. The colored bar height represents the mean of the measurements, with small bars above and below indicating the standard deviation. The animal numbers were assigned randomly prior to genotyping, and this served to blind the investigators until the grouping for statistical analysis. To ensure the fidelity of the *Adamts5^−/−^* homozygous phenotype *Adamts5^−/+^* het X *Adamts5^−/+^* het matings were performed; this approach also generated *Adamts5^−/−^* and *Adamts5^+/+^* littermate controls. The number of mice utilized in this study represents a minimum of 10 different litters per timepoint to obtain an appropriate n for this study for each developmental stage analyzed. 

## 3. Results

### 3.1. The Pulmonary Valves of Adamts5^−/−^ Mice Exhibited a Transient Phenotypic Rescue at Postnatal Day 7

**Figure 1:**
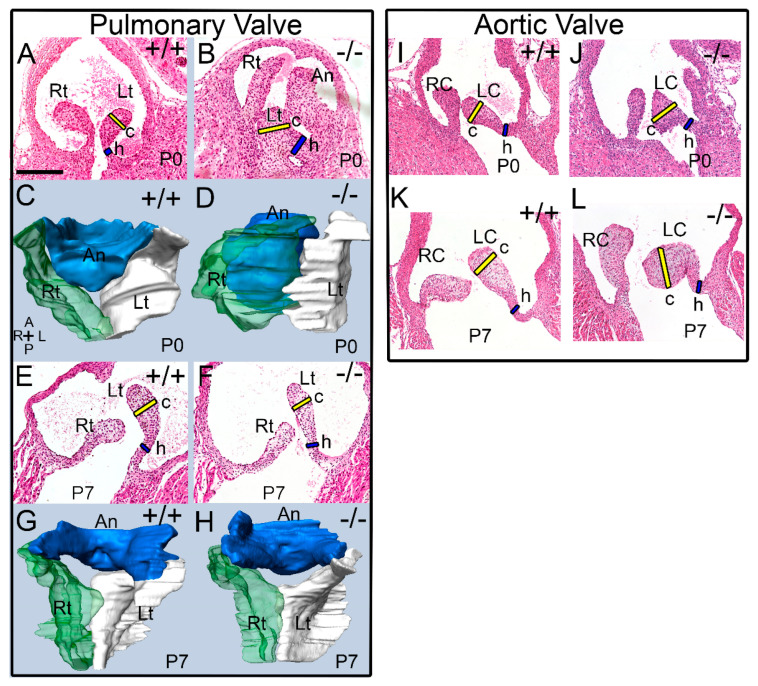
*Three-dimensional reconstructions and histological sections revealed that the abnormal enlarged morphology of the Adamts5^−/−^ pulmonary valves at birth was transiently corrected at postnatal day 7.* Histological sections (**A**,**B**,**E**,**F**) and three-dimensional (3D) reconstructions (**C**,**D**,**G**,**H**) indicate the morphological correction of the *Adamts5^−/−^* pulmonary valve (PV). Hematoxylin and Eosin-stained (**H** & **E**) sections show the normal morphology of the wild type (+/+; **A**) PV compared to the *Adamts5^−/−^* PV (−/−; **B**) at postnatal day 0 (P0). 3D reconstructions at P0 revealed the enlarged, block-like shape of the *Adamts5^−/−^* PV (**D**). Rt—right cusp of the PV, Lt—left cusp of the PV, An—anterior cusp of the PV; RC—right coronary cusp of the aortic valve (AV), LC—left coronary cusp of the AV. At postnatal day 7, (P7) the normal sculpted morphology of the PV cusps of +/+ and −/− PV (**E**,**F**) at P7 was evident in the 3D reconstructions (**G**,**H**; Blue—An PV cusp, Green—Rt PV cusp, and White—Lt PV cusp). Black bar in **A** = 150 μm and applies to (**B**–**L**). Orientation to 3D reconstructions (**C**): A—Anterior, P—Posterior, R—Right, L—left. Blue bars on each (**H** & **E**) section indicates hinge region measurement; yellow bars indicate the area of the cusp that were measured, related data is in Figure 2. Histological sections and 3D reconstructions were representative of each genotype and timepoint.

We previously published that mice deficient in the ADAMTS5 protease have larger PV, AV, and MVs at embryonic day 17.5 (E17.5) and in adult mice (>6 mo) [14]. In this study, the PV and AV of *Adamts5^−/−^* mice at P0 (birth), P7, and 1 mo were analyzed. Histological sections and 3D reconstructions of the WT at P0 (n = 6) and P7 (n = 3) as well as *Adamts5^−/−^* PV at P0 (n = 7) and P7 (n = 4) were generated and analyzed (Figure 1). Histological comparisons revealed significant morphological correction of the *Adamts5^−/−^* enlarged valve cusps from P0 to P7 (Figure 1). Comparisons of PV 3D reconstructions from P0 (Figure 1D), and P7 *Adamts5^−/−^* mice (Figure 1H) highlighted the thinner and more symmetric valve cusps that were similar to WT at P7. The morphological changes in valve shape and size indicated an apparent rescue of the enlarged valve phenotype found in prenatal and P0 *Adamts5^−/−^* mice to the sculpted morphology observed in WT.

**Figure 2:**
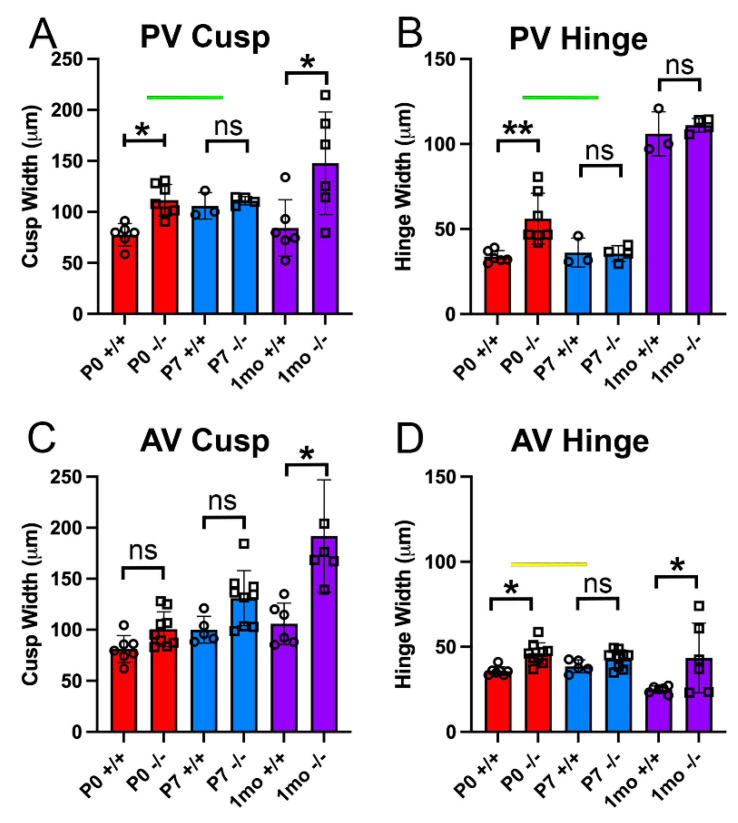
*The widths of the Adamts5^−/−^ pulmonary valve cusp and hinge regions were indistinguishable from wild type at postnatal day 7, indicative of a phenotypic rescue.* The thickness of the pulmonary valve (PV) cusp (**A**), PV hinge (**B**) aortic valve (AV) cusp (**C**) and AV hinge (**D**) were quantified at postnatal day 0 (P0, red), postnatal day 7 (P7, blue) and 1 month of age (1 mo, purple) in the wild type (+/+) and *Adamts5^−/−^* (−/−) mice. Green bars-denote the transient loss of PV *Adamts5^−/−^* cusp phenotype (**A**) and hinge phenotype (**B**) from P0 to P7. Yellow bar—denotes the transient correction of the hinge regions of the AV. Open circles on each graph-wild type; open squares- *Adamts5^−/−^*. *—*p* < 0.05; **—*p* < 0.01; ns—not significant. Each symbol on the graph denotes data from one mouse.

Quantification of the cusp and hinge regions of the PV and AV of *Adamts5^−/−^* at P0 (n = 7), P7 (n = 4), and 1 mo (n = 6) as well as WT mice at P0 (n = 6), P7 (n = 3), and 1 mo (n = 6), revealed that the PV exhibited a significant transient rescue of the enlarged phenotype and was indistinguishable from WT littermates at P7 (Figure 2A,B). Although the *Adamts5^−/−^* mice exhibited a significantly enlarged PV throughout embryological development, including P0, by P7 there was no significant difference in the *Adamts5^−/−^* PV cusp (Figure 2A) or hinge regions (Figure 2B) compared to WT. However, once the *Adamts5^−/−^* mice reached 1 mo of age, the PV cusps were significantly enlarged (Figure 1), indicating that the ‘rescued’ phenotype was transient. In contrast, the *Adamts5^−/−^* AV exhibited different morphological dynamics than the PV. The AV cusps of *Adamts5^−/−^* mice (n = 9) were not significantly larger than WT (n = 7) at P0 or P7 (*Adamts5^−/−^*, n = 9; *Adamts5^−/−^*, n = 5; Figure 1I–L; Figure 2C,D; (P0-*p* = 0.306; P7-*p* = 0.332). Of note the *Adamts5^−/−^* hinge regions of the PV and AV also differed in their phenotypic progression (Figure 2B,D). There were no significant differences in the AV cusps between P0 and P7, i.e., no ‘AV phenotypic rescue’ (Figure 2C,D; *p* = 0.332; (WT, n = 5), (*Adamts5^−/−^*, n = 9)). However, as postnatal development progressed to 1 mo, both the AV and PV cusps from *Adamts5^−/−^* mice (n = 5) were significantly larger than WT (n = 6; *p* < 0.020). 

### 3.2. Proteoglycan Proteases ADAMTS1, MMP2, and MMP9 Were Present in Postnatal Cusp and Hinge Regions of the Pulmonary Valve

We reasoned that the transient phenotypic correction of the *Adamts5^−/−^* PV may be due to proteolytic cleavage and clearance of VCAN from proteoglycanases other than ADAMTS5 that were upregulated at birth in the *Adamts5^−/−^* mice. 

#### 3.2.1. ADAMTS1 Is the Closest Family Member to ADAMTS5, Exhibits Catalytic Activity to VCAN, and May Compensate for the Loss of ADAMTS5 from P0 to P7

**Figure 3:**
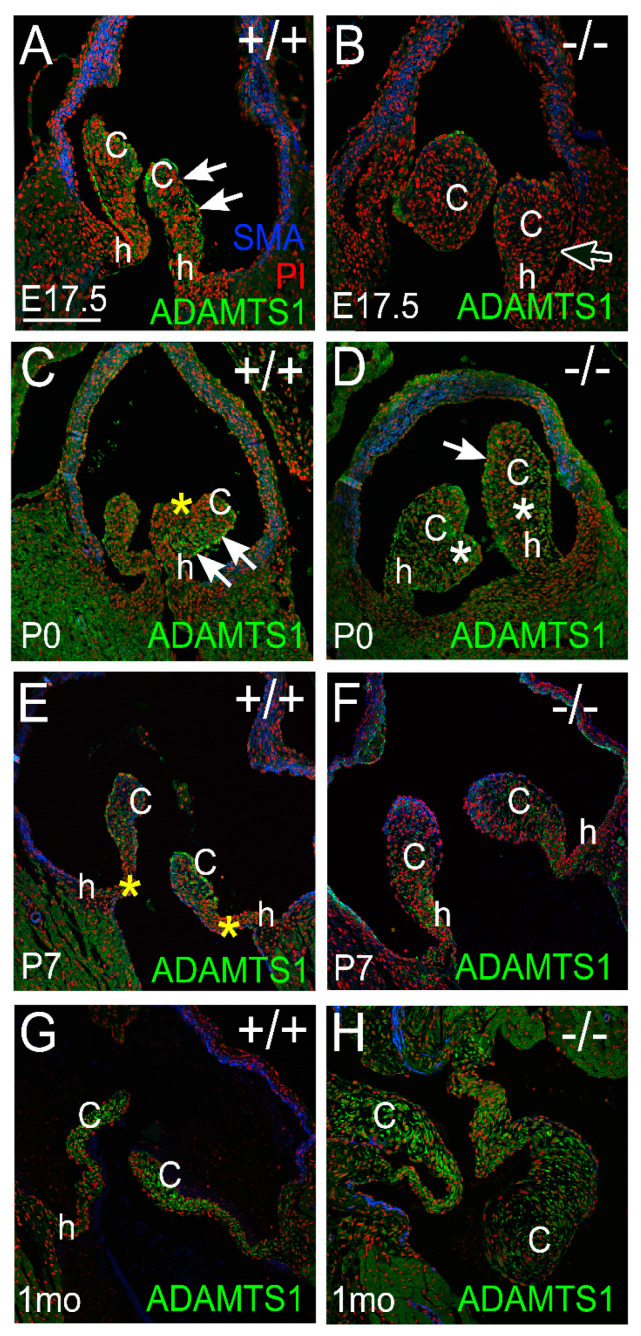
*ADAMTS1 localization is evident in a subset of valve ECM and is increased after birth in the Adamts5^−/−^ pulmonary valve.* ADAMTS1 immunolocalization (green) at embryonic day 17.5 (E17.5; **A**,**B**), postnatal day 0 (P0; **C**,**D**), postnatal day 7 (P7; **E**,**F**) and 1 month of age (1 mo; **G**,**H**) in wild type PV (+/+; **A**,**C**,**E**,**G**) and *Adamts5^−/−^* PV (−/−; **B**,**D**,**F**,**H**). c—valve cusp; h—valve hinge region. White asterisk (**D**), increased localization in cusp; white outline arrow (**B**)—loss of ADAMTS1 in the endocardium; Red—propidium iodide; Blue—αSMA (used as a marker of smooth muscle cells in the arterial wall.) Solid white arrows—endocardial expression of ADAMTS1; yellow asterisk—layer of cusp devoid of ADAMTS1 localization. Bar in (**A**) = 150 μm and applies to (**B**–**H**).

ADAMTS1 was observed in PVs at late stages of valve maturation. Images are representative of n = 3 for each genotype and at each stage. At E17.5, there was prominent staining of ADAMTS1 in the endocardium of WT PV cusps with localization to the arterial side (Figure 3; white arrows). At E17.5, the enlarged cusps in the *Adamts5^−/−^* PV exhibited reduced ADAMTS1 localization compared to WT (Figure 3B; white outline arrow). However, shortly after birth there was considerably more ADAMTS1 in the *Adamts5^−/−^* PV cusp VICs than late-stage gestation (Figure 3D, white asterisks). At P0, the WT cusps exhibited a stratified expression of ADAMTS1, with predominant localization on the arterial side and undetectable expression on the ventrialis side (Figure 3C,D; white arrows, yellow asterisk). In WT cusps at P7, ADAMTS1 was expressed in the cusps but not in the hinge regions (Figure 3E, yellow asterisks), while the *Adamts5^−/−^* P7 cusps exhibited ADAMTS1 localization in both the cusp and hinge regions (Figure 3F). At 1 mo, ADAMTS1 was localized to the distal regions of the cusps in the WT but observed throughout the *Adamts5^−/−^* cusps (Figure 3G,H).

#### 3.2.2. MMP2 Was Evident in the Pulmonary Valve and May Play a Role in Late-Staged Valve Maturation

**Figure 4:**
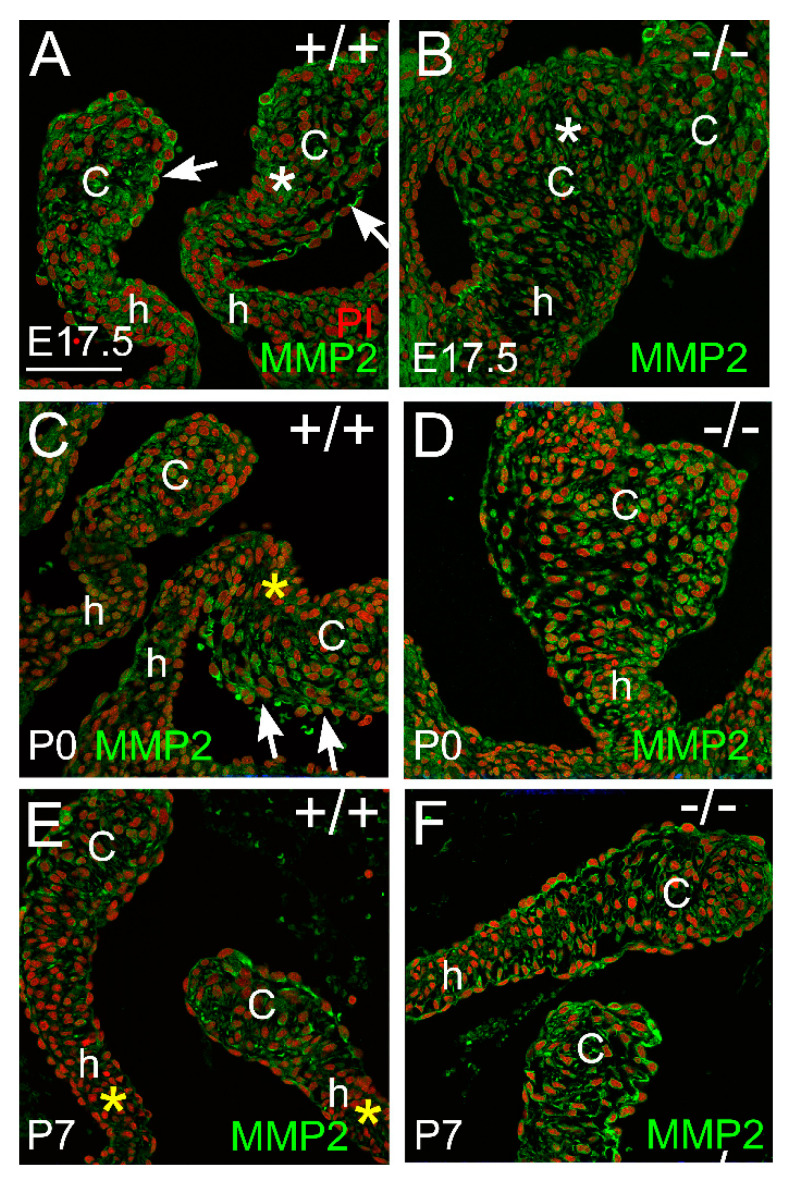
*MMP2 localization is present in the endocardium and valvular interstitial cells of developing cardiac valves.* MMP2 immunolocalization (green) at embryonic day 17.5 (E17.5; **A**,**B**), postnatal day 0 (P0; **C**,**D**), and postnatal day 7 (P7; **E**,**F**) in wild type (+/+; **A**,**C**,**E**) and *Adamts5^−/−^* PV (−/−; **B**,**D**,**F**). c—valve cusp; h—valve hinge region. Red—propidium iodide. Solid white arrows—endocardial expression of MMP2; white asterisk—localization of MMP2 throughout the VICs; yellow asterisk—region of VICs devoid of MMP2 localization. Bar in (**A**) = 150 μm and applies to (**B**–**F**).

MMP2 was localized within the endocardium and valvular interstitial cells of the cusps in late gestation (Figure 4A,B). MMP2 expression was stratified on the arterial side of the cusps shortly after birth in WT mice but was not observed in the ventricularis region (Figure 4C; white arrows and yellow asterisk). *Adamts5*^−/−^ cusps exhibited a more homogenous staining pattern of MMP2 in the cusp than WT (Figure 4D). At P7, the WT and the *Adamts5*^−/−^ cusps exhibited similar MMP2 localization in the endocardium and VICs. 

#### 3.2.3. MMP9 Was Localized in the Maturing Valve Cusps in Regions where the Mesenchymal Cells Were Highly Compacted

**Figure 5:**
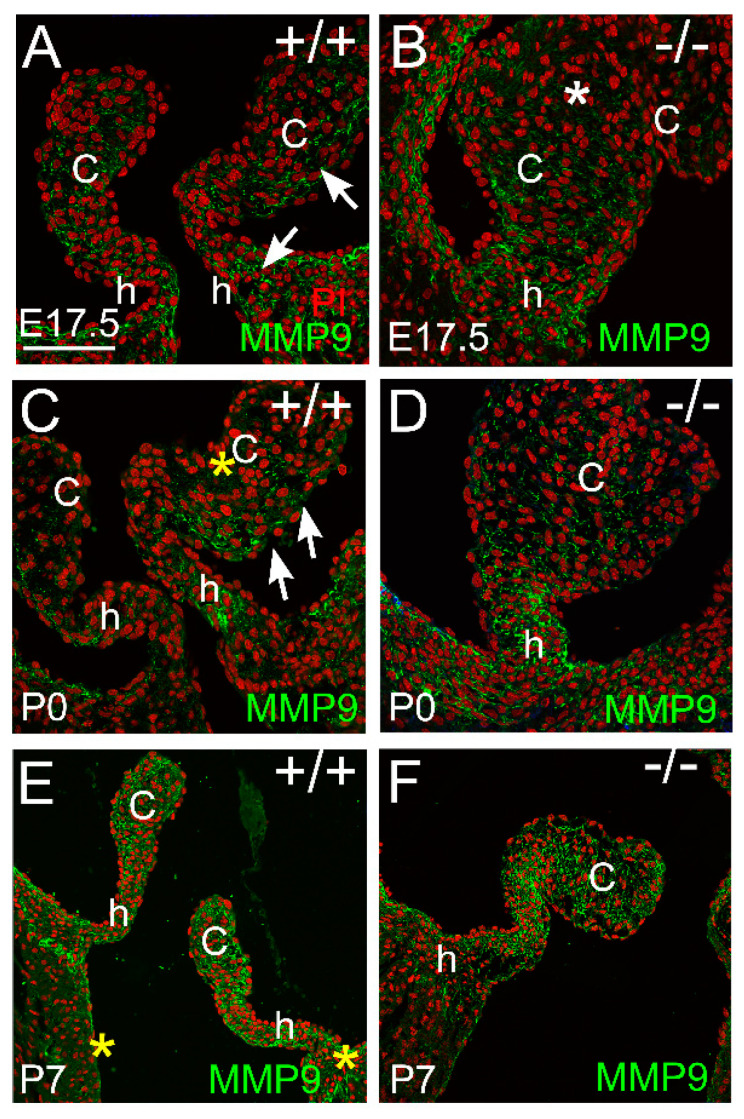
*MMP9 is localized in valvular interstitial cells of developing cardiac valve cusp and hinge regions.* MMP9 immunolocalization (green) at embryonic day 17.5 (E17.5; **A**,**B**), postnatal day 0 (P0; **C**,**D**), and postnatal day 7 (P7; **E**,**F**) in wild type (+/+; **A**,**C**,**E**) and *Adamts5^−/−^* PV (−/−; **B**,**D**,**F**). c—valve cusp; h—valve hinge region. Red—propidium iodide. White asterisk—localization of MMP9 throughout the VICs, yellow asterisk-region of VICs devoid of MMP9 localization. Bar in (**A**) = 150 μm and applies to (**B**–**F**).

At E17.5, MMP9 was localized in the PV hinge and cusp regions of the WT (Figure 5A, arrows) but MMP9 was not detected in the distal cusp regions of the *Adamts5*^−/−^ (Figure 5B, white asterisk) where the VICs are not as condensed as the WT [14]. At P0, MMP9 exhibited a stratified expression pattern in the WT cusps (Figure 5C, yellow asterisk-no staining, arrows-staining); and was localized in the hinge regions (Figure 5C, h); the *Adamts5*^−/−^ cusps exhibited homogeneous expression throughout the cusps and localization in the hinge regions (Figure 5D). At P7, the localization of MMP9 was similar in the hinge and cusp regions in WT and *Adamts5*^−/−^ PV (Figure 5E,F). 

### 3.3. Localization of the Small Leucine-Rich Proteoglycan Decorin Was Overlapping with Versican at P0 in the Adamts5^−/−^ Cusps, but at P7 DCN and VCAN Were Restricted to Separate Layers Consistent with ‘Phenotypic Rescue’

**Figure 6:**
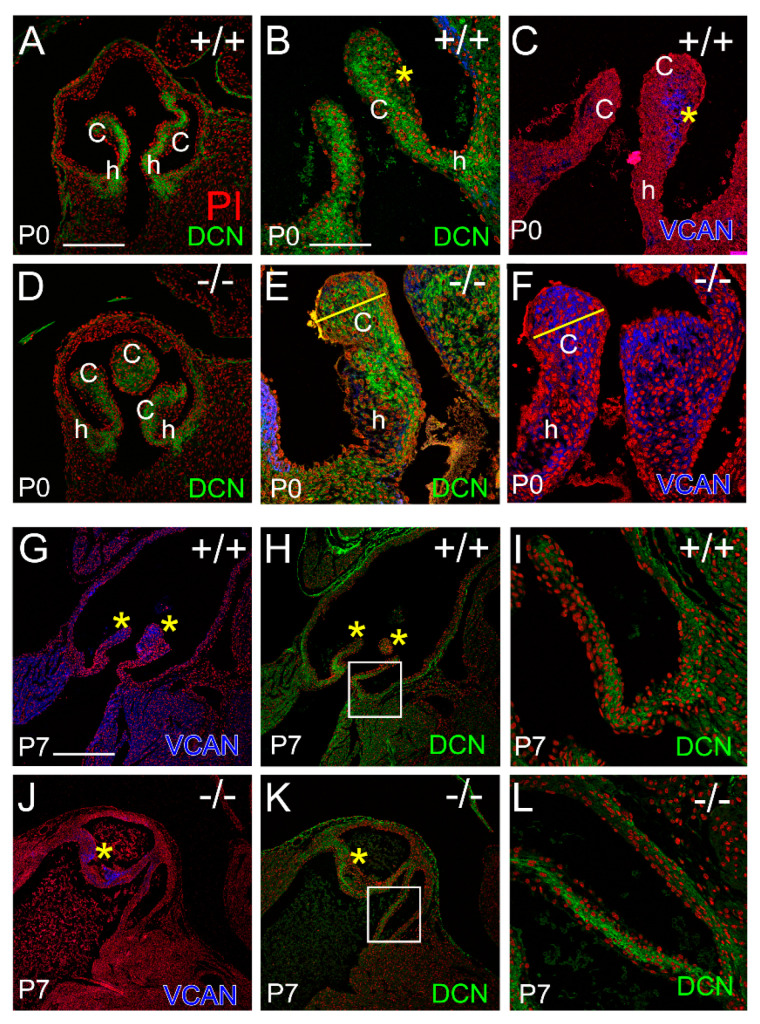
*DCN and VCAN exhibit stratified localization in the Adamts5^−/−^ PV at P7 that display a transient phenotypic rescue and similar to WT.* DCN localization (green) at postnatal day 0 (P0; **B**,**D**,**E**) and P7 (**H**,**I**,**K**,**L**) and VCAN (blue) at P0 (**C**,**F**) and P7 (**G**,**J**). c—valve cusp; h—valve hinge region. Yellow asterisks (**B**,**C**,**G**,**H**,**J**,**K**) indicate complementary expression patterns of DCN and VCAN while yellow lines (**E**,**F**) designate overlapping expression patterns of DCN and VCAN. Red-propidium iodide. Bar in (**A**) = 150 μm and applies to (**D**); Bar in (**B**) = 50 μm and applies to (**C**,**E**,**F**,**I**,**L**); Bar in **G** = 225 μm and applies to (**H**,**J**,**K**).

The Small Leucine Rich Proteoglycan (SLRP) Decorin (DCN), named because it decorates Collagen I, was evident in the venticularis of the cusps, as well as the anchor and hinge regions by P0 in both the WT and *Adamts5^−/−^* PV (Figure 6A–F). The organization of DCN at P0 in the PVs appeared more prominent in the WT (Figure 6A,B) than *Adamts5^−/−^* (Figure 6D,E). In the WT PV at P0, the immunolocalization of DCN (Figure 6B, green, yellow asterisk) and versican (VCAN) (Figure 6C, blue, yellow asterisk) exhibited a complementing pattern while in the *Adamts5^−/−^* PV at P0, DCN and VCAN were overlapping (Figure 6E,F, yellow line). However, at P7, the organization of DCN in the *Adamts5^−/−^* PV was similar in the WT (Figure 6G–L); i.e., there was a distinction in the ECM layers of DCN and VCAN expression in the WT (Figure 6G,H, yellow asterisks) as well as the *Adamts5^−/−^* PV at P7 (Figure 6J,K, yellow asterisks). 

The immunolocalization of DCN and VCAN indicated that the mature fibrosa and spongiosa layers, respectively, were distinct at P7 in both the WT and *Adamts5^−/−^* PV which correlated with the P7 phenotypic rescue of the enlarged *Adamts5^−/−^* PV. 

## 4. Discussion

**Figure 7:**
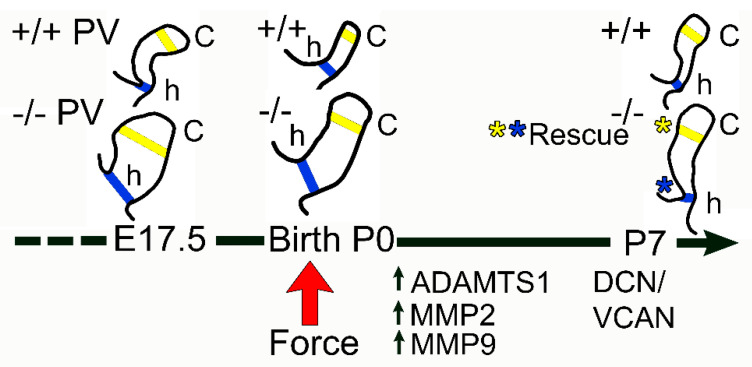
*Schematic depicting the transient morphological rescue of the pulmonary valve in Adamts5^−/−^ mice after birth.* Timeline of late prenatal and early postnatal phenotype of the pulmonary valve (PV), at embryonic day 17.5 (E17.5), birth (P0), and postnatal day 7 (P7). The schematic shows the left cusp of the PV drawn to relative scale within timepoints to compare the *Adamts5^+/+^* (+/+; wild type, WT) and *Adamts5^−/−^* (−/−) PVs. Yellow lines indicate width measurements of the cusps while blue lines indicate the width measurements of the hinge regions. At P7, Both the cusp and the hinge regions are reduced in the −/− cusps and similar to +/+ (asterisks yellow and blue, respectively). Red arrow indicates birth with onset of blood flow to the lungs. ADAMTS1, MMP2, and MMP9 exhibited expanded localization profiles in the −/− PV after birth. The rescued *Adamts5^−/−^* PV also exhibited complementary localization of DCN and VCAN which had overlapping expression at E17.5 and birth.

In this study, we investigated ECM protease–substrate localization in the early postnatal timeframe where biomechanical forces are altered due to the initiation of blood circulation to the lungs. The fully penetrant enlarged PV cusp and hinge phenotypes in the *Adamts5^−/−^* mice were resolved to a sculpted appearance indistinguishable from WT at P7. This apparent phenotypic rescue of the *Adamts5^−/−^* PV may be the result of increased ECM proteoglycanase activity in the VICs and endocardium after birth; of note, these proteoglycanases were reduced in the *Adamts5^−/−^* PV compared to WT prior to birth. ADAMTS1, MMP2, and MMP9, that also cleave VCAN, may compensate for the loss of ADAMTS5 by decreasing VCAN levels thereby restoring normal PV morphology. The apparent increase in proteoglycanase activity at birth in the PV may be the result of increased blood flow to the lungs. It is likely that ADAMTS-VCAN-substrate interactions are activated by mechanotransducive pathways that drive cardiac valve maturation (Figure 7).

Mechanosensors of shear force such as Krüppel-like factor 2 (Klf2/4) and endothelial nitrous oxide synthetase are expressed by endocardial cells of developing valves and their genetic perturbation in mice disrupts cardiac valve development [32,33,34,35,36]. Proteoglycanases ADAMTS5 [14], ADAMTS1 [37], and MMP2 are localized to the valvular endocardium, where shear and compressive forces are generated. Moreover, ADAMTS-cleaved VCAN fragments are found in the endocardial ECM as well as the adjacent VICs [14]. ADAMTS5 interacts with Klf2/4 as part of the endothelial cell cerebral cavernous malformation pathway which, when disrupted, leads to over-expression of *Adamts5* and premature loss of provisional cardiac valve ECM (cardiac jelly) with reduced proliferation of adjacent myocardial cells. Loss of endocardial expression of *Adamts19* also perturbs shear stress signaling and leads to increased mesenchymal cells and proteoglycan deposition in cardiac valves [26]. Klf2 together with *Adamts5* are upregulated in the *Adamts19^−/−^* valvular endocardium [26]. It is not clear if *Adamts5* and *Adamts19* are direct targets of the mechano-induced Klf2, but they are integrated within mechanosenstive pathways localized to the endocardium. 

During valve development, mechanical signals are transmitted from the endocardium to the underlying VICs, in part through incorporation of nitrous oxide. Mechanosensitive ion channels also regulate endothelial response to shear flow [38,39]. By mid-gestation, a subset of VICs, adjacent to the endocardium, is differentiated from others by their condensed cell behavior, as well as localization of cytoskeletal proteins, αSMA and Filamin A [16]. This subset of VICs is devoid of intact VCAN, but resides in an ECM of cleaved VCAN (referred to as DPEAAE or Versikine) [14]. Deletion of ADAMTS5 with loss of cleaved VCAN results not only in the disruption of VIC condensation, but also loss of αSMA, and Filamin A localization [16]. Since the actin cytoskeleton is highly responsive to changes in mechanical forces [40,41], this further implicates the ADAMTS5-VCAN, protease–substrate pair as an essential component of mechanically responsive pathways during cardiac valve development. The mechanisms by which cleaved VCAN facilitates a cytoskeletal response, and/or increased VCAN inhibits the cytoskeletal organization, will require further investigation.

The limitations of this study include the assumption that mechanical load changes on the PV when blood circulation to the lungs is initiated at birth. Proteoglycans are also regulated by transcription, not evaluated here. ADAMTS and MMP activity is inhibited by TIMP3 and not investigated in this study. While the known repertoire of substrates for the ADAMTS proteoglycanases is limited, our understanding of their role in development and disease will be significantly advanced as additional in vivo substrates are identified [7,42,43].

## 5. Conclusions

This study indicates that proteolytic cleavage of proteoglycans may be induced by altered mechanical load to promote ECM maturation during cardiac valve development. In the murine model of ADAMTS5 deficiency, loss of this protease results in enlarged cardiac valves that contain excess VCAN; the increased localization of other proteoglycanases in the maturing valve cusps corrects the morphological defect by P7 and correlates with ECM organization, specifically VCAN in the spongiosa and DCN in the Collagen I-rich fibrosa layer. This study indicates that proteoglycanase activity may be an essential downstream mechanism of mechanotransduction that is important for normal cardiac valve maturation. Since increases in proteoglycans are evident in diseased cardiac valve tissues, understanding mechanisms that reduce proteoglycan content is essential for developing effective therapeutic strategies to treat cardiac valve diseases. 

## Data Availability

Not applicable.
